# Mitochondria as the target for disease related hormonal dysregulation

**DOI:** 10.1016/j.bbih.2021.100350

**Published:** 2021-09-21

**Authors:** Gladys A. Shaw

**Affiliations:** Department of Anatomy and Neurobiology, Virginia Commonwealth University, Richmond, VA, USA

**Keywords:** Mitochondria, Estrogen, Aging, Stress, Inflammation, Neurodegeneration

## Abstract

Mitochondria play an important role in the synthesis of steroid hormones, including the sex hormone estrogen. Sex-specific regulation of these hormones is important for phenotypic development and downstream, sex-specific activational effects in both brain and behavior. First, mitochondrial contribution to the synthesis of estrogen, followed by a discussion of the signaling interactions between estrogen and the mitochondria will be reviewed. Next, disorders with an established sex difference related to aging, mood, and cognition will be examined. Finally, review of mitochondria as a biomarker of disease and data supporting efforts in targeting mitochondria as a therapeutic target for the amelioration of these disorders will be discussed. Taken together, this review aims to assess the influence of E2 on mitochondrial function within the brain via exploration of E2-ER interactions within neural mitochondria and how they may act to influence the development and presentation of neurodegenerative and neurocognitive diseases with known sex differences.

## 1 Synthesis of sex steroid hormones in the mitochondria

Mitochondria play a crucial role many cellular functions, acting as the primary manufacturing site of adenosine triphosphate (ATP) and primary site of synthesis for steroid hormones in the brain, adrenal glands, gonads, and other serotonergic organs (Velarde, 2014), as well as a regulator of cellular calcium homeostasis (Klinge, 2017). Mitochondria house the rate limiting enzymes necessary in the creation of steroid hormones (Gavrilova-Jordan and Price, 2007; Sanderson, 2006; Velarde, 2014). This includes the sex steroid hormone estrogen, which is created through enzymatic mechanisms within the mitochondria of the adrenals, gonads, and brain (Sanderson, 2006).

Mitochondria are not only the initial site of steroidogenesis, but also use sex steroid hormones as modulatory factors for its own function and gene transcription (Miller, 2013; Sanderson, 2006; Velarde, 2014). Due to the dynamic nature of mitochondria, the changing cellular environment caused by external stimuli alter both the localization (Fang et al., 2012) and structure (Flameng et al., 1980; Rossi and Pekkurnaz, 2019) of the mitochondria within the cell. Hormones are no exception to this rule. Sex steroid hormone production is both a regulator and is regulated by mitochondrial activity and functionality. The sex steroid hormones, including estrogen, directly act upon mitochondria through the non-classical pathway (Del Río et al., 2018), as its direct point of action is not within the nucleus but rather the mitochondria itself. Receptors for these hormones have been found at varying levels in the mitochondria of both males and females (Picard and McEwen, 2014; Velarde, 2014).

## 2 Estrogen modifies mitochondrial activity

The interaction between estrogen and the mitochondria is the most studied of any of the sex steroid hormones. Three estrogen variants exist in females; estrone (El), 17ß-estradiol (E2), and estriol (E3), with E2 being the primary form of estrogen in circulation (Rettberg et al., 2014). A large amount of estrogen in the female body is released from the ovaries; the female gonadal organs. When released from the ovaries, estrogen circulates throughout the body, and crosses the blood-brain barrier to act upon the cells within the brain (Rettberg et al., 2014). In males, the primary source of estrogen is maintained through the conversion of testosterone to estrogen via the enzyme aromatase (Chen et al., 2005; Gervais et al., 2019; Saldanha et al., 2009). This process also occurs in females, but to a lesser extent (Cornil, 2018; Roselli and Klosterman, 1998; Stanić et al., 2014). In both cases, estrogen is able to interact with neural mitochondria, eventually reaching and binding to both estrogen receptors (ER) and estrogen response elements (ERE) within the organelle (Arnold and Beyer, 2009; Chen and Yager, 2004; Rettberg et al., 2014).

**Fig. 1:**
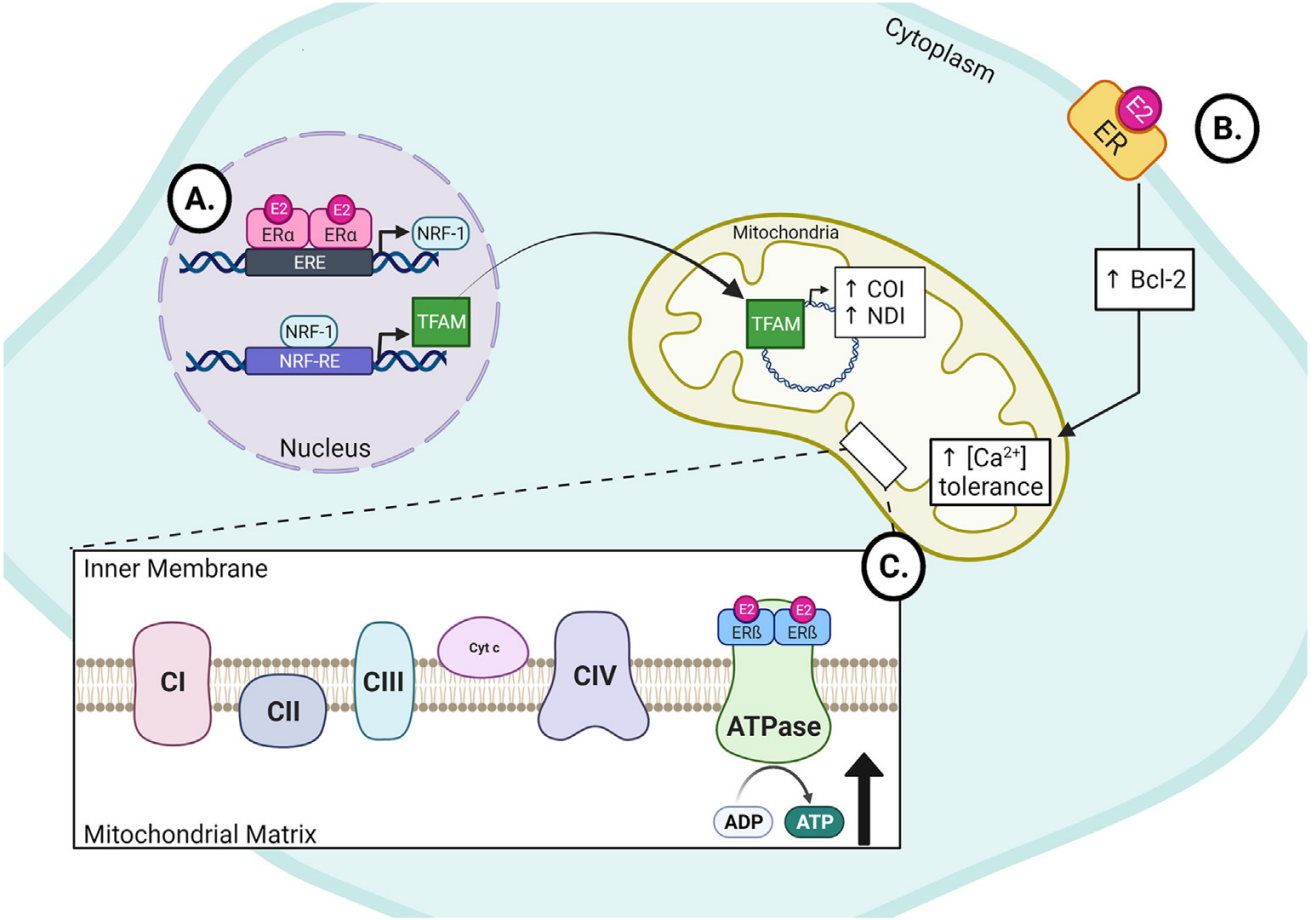
Simplified mechanism of mitochondria-estrogen interactions. A) Estrogen receptor ​α ​(ERα) acts as a transcription factor within the nucleus to promote the expression of NRF-1. Subsequently, NRF-1 acts to upregulate the transcription of TFAM, which is then sent to the mitochondria to act as a transcription factor within the mitochondrial DNA. This action increases the mitochondrial production of COI and NDI, proteins that increase mitochondrial respiration and biogenesis (Mattingly et al., 2008). **B)** ​Interactions between estrogen (E2) and the membrane bound estrogen receptor (ER) act to upregulate Bcl-2 expression, an anti-apoptotic protein. The downstream activity of Bcl-2 increases the calcium buffering capabilities of the mitochondria, preventing cellular death (Nilsen and Brinton, 2003). **C)** ​Direct interactions between E2 and estrogen receptor ​ß ​(ERß) act within the mitochondria to increase the production of ATP via the ATPase ​(Álvarez-Delgado et al., 2010, Irwin et al., 2008, Liao et al., 2019). It is worth noting that estrogen has been found to modulate the activity of complexes I, III, and IV as well (not shown,(Lejri et al., 2018)). Figure created using ​BioRender.com.

To date, three ERs have been characterized in animals. ERα and ERß are found in all mammalian tissues (Adzic et al., 2017; Liao et al., 2015; Simpkins et al., 2008). It is worth noting the existence of a third receptor, ERγ, found at relatively lower levels in fish (Hawkins et al., 2000), yet there is no evidence of this receptor in mammals (Amenyogbe et al., 2020; Rettberg et al., 2014). The receptors are highly localized, with reported evidence suggesting ERß is the primary receptor found within the mitochondria of neurons (Herrick et al., 2006; Mehra et al., 2005; Milner et al., 2005; Shimamoto and Rappeneau, 2017; Yang et al, 2004, 2009). ERα also plays a significant role in estrogen mediated mitochondrial activity through nucleus-mitochondria crosstalk (Arnold and Beyer, 2009; Rettberg et al., 2014). In a study by Mattingly et al., (2008), their data displayed the role of ERα in mitochondrial function. They revealed *in vitro* E2 treatment increased the transcript and protein levels of nuclear respiratory factor 1 (NRF-1) via E2-ER interactions within the nucleus. This is particularly interesting as NRF-1 is a nuclear transcription factor that regulates the expression of the mitochondrial transcription factor A (TFAM), a nuclear encoded mitochondrial gene that controls transcription of mitochondrial DNA (mtDNA) (Mattingly et al., 2008). The rise in TFAMs promotes the transcription of cytochrome *c* oxidase I (COI) and NADH dehydrogenase I (NDI) from mtDNA, both acting to increase mitochondrial biogenesis and respiratory activity (Fig. 1A). Together, these data a support the claim that estrogen influences the preservation of mitochondrial respiration, once again, suggesting a neuroprotective mechanism mediated by estrogen.

Membrane bound ERs have been shown to influence the mitochondria's ability to utilize calcium. Nilsen and Brinton report that hypothalamic neurons treated *in vitro* with E2 enhanced levels of anti-apoptotic Bcl-2 proteins within their mitochondria when exposed to glutamate through interactions with an unspecified membrane bound ER (Nilsen and Brinton, 2003; Fig. 1B). Conversely, the mitochondria of aromatase-knockout male mice are shown to have altered mitochondrial Bcl-2 levels, inhibited mitochondrial calcium permeability, and increased cardiolipin levels that is rescued with E2 supplementation (Moro et al., 2010). Although Moro et al. used mitochondria from the liver and not the brain and did not assess cognition, a study by Burstein et al. (2018) found the neural mitochondria of female mice display increased sensitivity of the mitochondrial calcium permeability than that of males, likely due to ERß (Burstein et al., 2018). Together, these data suggest a neuroprotective role of estrogen. This ultimately occurs through mitochondrial regulation within neurons, as Bcl-2 increases the calcium buffering ability of mitochondria, subsequently preventing apoptotic signaling.

EREs directly interact with both ERα and ERß (Rettberg et al., 2014) and have been shown to exhibit a positive correlation with E2 and mitochondrial ER (mtER) binding affinity. ERE activity within mtDNA acts to regulate genes within the promoter region of mtDNA. Thus, estrogen not only acts directly on gene transcription, but also has indirect activity through the enhancement of mtDNA gene promoters.

Estrogen has also been shown to play a role in the regulation of mitochondrial function, aerobic respiration, and glucose transport (Arnold and Beyer, 2009; Del Río et al., 2018; Rettberg et al., 2014). In a study using HepG2 and MCF-7 ​cells, data supports the claim that ERs within the mitochondria, when bound to E2, act as transcription factors through their activity with mitochondrial EREs (Chen and Yager, 2004). Although the direct action of ER-ERE complexes alter transcript levels within mtDNA *in vitro*, there is evidence that this occurs *in vivo* within the brain as well (Rettberg et al., 2014). Additionally, there has been evidence of the direct activity of E2 and ATP production. Previous findings support the idea that E2 directly interacts with the electron transport chain (ETC) through direct interaction and modulation of the activity of the ATPase through ERß (Álvarez-Delgado et al., 2010; Chen et al., 2009; Del Río et al., 2018; Irwin et al., 2008; Liao et al., 2019; Fig. 1C).

Evidence of this phenomenon is supported by an experiment by Alverez-Delgado and colleagues showing coimmunoprecipitation of the ATPase and ERß from whole brain protein isolates (Álvarez-Delgado et al., 2010). This is thought to occur through modulation of glucose availability within the cell, subsequently altering the rate at which ATP is produced (Irwin et al., 2008). Moreover, *in vitro* upregulation of mitochondrial ERß increases both ATP synthesis, whereas application of an ERß antagonist not only decreased mitochondrial respiration, but also decreased the production of ATP (Liao et al., 2019). Taken together, these data support the claim that estrogen mediates mitochondrial activity indirectly through E2 interactions with the nuclear ERα and directly through transcriptional regulation of mtDNA and availability of substrates necessary for ATP synthesis via mitochondrial ERß.

## 3 Hormonal influence of mitochondria-driven neuro-anatomic and behavioral changes

The lifelong impact of the ever-modulating levels of sex hormones in the development of disease are most clearly seen in the incidence of disorders traditionally associated with stress and aging. Specifically, the incidence of Alzheimer's disease (Lindemer et al., 2018; Nemeth et al., 2013), anxiety (Altemus et al., 2014), and depression (Bekhbat and Neigh, 2018; Fan et al., 2019; LeWinn et al., 2014; Tham et al., 2011) are higher in women than that in men and have been associated with altered estrogen levels (Mohajeri et al., 2019). The role of mitochondria in these and other neurodegenerative and neurocognitive disorders are of increasing interest, as the modern theory is that these pathologies are driven by mitochondrial malfunction (Chang et al., 2019; Hoffmann and Spengler, 2018; Marazziti et al., 2012; Shimamoto and Rappeneau, 2017).

### 3.1 Alzheimer’s disease

Alzheimer's disease (AD) is the most common neurodegenerative disease, with approximately 6.1 million adults over the age of 65 years old with clinical AD in the United States as of 2020 (Rajan et al., 2021). This disorder is characterized by both neuronal death and dysregulation of synapses in brain regions related to learning and memory (Mattson et al., 2008), most notably through the increase in amyloid beta (Aß) and hyperphosphorylation of tau (Grimm et al., 2016; Lezi and Swerdlow, 2012; Pickett et al., 2018). Dysfunctional mitochondria are one of the first signs of the disorder, appearing decades before official diagnosis (Marazziti et al., 2012; Olesen et al., 2020; Song et al., 2021; see Song et al., 2021 for review). Both Aß and tau accumulation involve altered glucose utilization (Mattson et al., 2008; Weidling and Swerdlow, 2019), decreased production of ATP (Grimm et al., 2016; Mattson et al., 2008), increased mitochondrial O_2_ production (Lezi and Swerdlow, 2012; Mattson et al., 2008; Wang et al., 2019), and an increase in mitochondrial calcium uptake (Marazziti et al., 2012; Mattson et al., 2008; Wang et al., 2019).

Decreased cytochrome C oxidase is a common mitochondrial deficit seen in both blood and brain from AD patients (Mattson et al., 2008; Weidling and Swerdlow, 2019). The transcription of mitochondrial cytochrome C oxidase is mediated by TFAM expression and has been shown to have a positive correlation with mitochondrial oxidative phosphorylation (Nilsen and Brinton, 2004). This suggests that a key event in the progression of AD, as well as other disorders involved in chronic neurodegeneration, is through the impairment of the major roles of mitochondria; energy production, calcium sequestration, and apoptotic signaling (Lezi and Swerdlow, 2012; Marazziti et al., 2012; Mattson et al., 2008; Mohajeri et al., 2019), though this statement remains a topic of debate (Weidling and Swerdlow, 2019). Synaptic mitochondria are specifically impacted by AD pathology (Olesen et al., 2020). Both mitochondrial function and transport are hindered by tau and Aß, with evidence of a fourfold reduction in mitochondria within the presynaptic terminals in Broadman's area 41 and 42, areas heavily targeted by AD neurodegeneration (Pickett et al., 2018). This suggests pinpointed alteration in mitochondrial dysfunction giving rise to the specific, learning and memory related deficits of the disease.

The influence in sex steroid hormones and AD development reveals a strong connection between hormone concentration, mitochondrial function, and behavioral changes. Notably is the connection between E2 and the prevalence of AD in females. Recent data has implied the decrease in ERß enhances the vulnerability to Aß cytotoxicity in both mice and humans (Levinta and Mukovozov, 2016; Uddin et al., 2020). Conversely, upregulation of ERß in rats protects against Aß via interactions with the Bcl-2 associated death promoter, with evidence suggesting this occurs independent of estrogen binding (Uddin et al., 2020). Together, these data suggest a major role of estrogen signaling and ERß in mitochondrial regulation in the development of AD.

### 3.2 Depression and anxiety

Sex differences in the incidence of the stress-induced neuropsychiatric disorders depression and anxiety.

(Heim et al., 2008; Wulsin et al., 2016; Yohn and Blendy, 2017) start to arise in adolescence (Paus et al., 2008; Shaw et al., 2020a), suggesting the role of sex steroid hormones in its emergence. Likewise, hormonal involvement in the development of anxiety-like symptoms beyond adolescence is strong, particularly in the hormonal drop that occurs postpartum (Magon and Kumar, 2012) and with reproductive senescence (Meethal and Atwood, 2005). Although the disorders often appear together, it is important to note that they are in fact different disorders with their own unique symptoms and challenges.

The role of hormones and mitochondrial dysfunction in the development of anxiety is robust, with hormonal changes and mitochondrial dysfunction seen in both human and rodent models (Shimamoto and Rappeneau, 2017). A sex-specific shift in mitochondrial respiration measured from synaptosomes of C56Bl/6 mice was found in females with a history of chronic repeated adolescent stress (CRPS), but not in males, despite showing equivalent increases in anxiety-like behavior in the open field (Shaw et al., 2020b). Moreover, subsequent injections with lipopolysaccharide (LPS) decreased synaptosomal respiration in both males and females who had undergone CRPS and chronic LPS treatment. Analysis of mitochondrial phenotype reveals a shift in the number of agranular and inflamed mitochondria following CRPS and LPS in females, whereas increases in the reactive oxygen species (ROS) protein ROMO-1 were seen only in males. Finally, females with a history of CRPS had significant alterations in protein leak related to the mitochondrial uncoupling in this study sample.

The decrease in transcription of the mitochondrial genes UCP2 and Bcl-2 have been associated with increased anxiety in human women (Shimamoto and Rappeneau, 2017), which was alleviated with hormone replacement therapy. Moreover, in a rodent study, an increase in testosterone levels have been shown to decrease fear and anxiety-like behaviors in female and male mice respectively (Celec et al., 2015). This group asserts that aromatase activity and interactions between testosterone metabolites with both the androgen and estrogen receptor play a major role in the behavioral and mitochondrial deficits seen in this disorder (Celec et al., 2015; Frye et al., 2008). Evidence suggests mitochondrial dysfunction related to ETC complex 4 and complex 5 activity led to increased ROS production that is attenuated following a combined supplementation of estrogen and progesterone. Therefore, it is plausible to assume the mtER and the progesterone receptor may be working together in a low-testosterone environment to give rise to an increased incidence of anxiety-like behaviors (Del Río et al., 2018; Irwin et al., 2008). This not only explains the behavioral changes in anxiety but also the mitochondrial deficits that are potential prodromal drivers of an anxiety-like phenotype.

Although clinical data displaying an explicit connection between depression, altered mitochondrial function, and E2 is comparatively sparse, rodent and *in vitro* data suggest the connection between the role of these three in the development of the depression and depressive-like behaviors (Shimamoto and Rappeneau, 2017). In rodents, low levels of estrogen have been associated with depressive-like behaviors (Shimamoto and Rappeneau, 2017). This finding is related to the inconsistent progression through the estrus cycle in rodents with a depressive-like phenotype. Here, the role of the mtER is a likely culprit in the development of depressive-like behavior. Data has shown that in ERß knockout mice, supplementation with estrogen has no change in depressive like behaviors, yet stimulation of the receptor increases these behaviors (Rocha et al., 2005; Walf and Frye, 2006). As ER activity has been shown to alter mtDNA transcription, a human study found that a decrease in the transcript levels of the mtDNA genes ND2, ND6, and CO2 correlates to an increased rate of depressive symptoms (Hoffmann and Spengler, 2018). Further research should be done to discern the exact mitochondrial modulations that may trigger the onset and behaviors accompanying depression in both basic and translational research.

## 4 Mitochondria as therapeutic targets

Mitochondria have been a topic of discussion for the development of new, targeted treatments. Pharmaceuticals targeting mitochondrial function, termed 'mitoceuticals' (Yonutas et al., 2020), are rapidly becoming the future of translational mitochondrial research. Innovations in mitochondrial transplantation have shown promising results in central nervous system related injury repair (Chang et al., 2019). Likewise, data from basic science are showing increasing evidence of mitochondrial dysfunction as a biomarker for various immune- (Chowdhury et al., 2010; Palavicini et al., 2020; Raoof et al., 2020) and hormone-related disorders (Głombik et al., 2020; Jeanneteau and Arango-Lievano, 2016; Picard et al., 2018; Zachariah et al., 2009). This section will focus on these advancements shaping the future of mitochondrial research generally and in relation to estrogen-mitochondria interactions.

### 4.1 MitoNEET

MitoNEET, a protein located on the outer membrane of mitochondria, was first identified in 2004 by Colca et al. In this groundbreaking study, Colca and colleagues revealed a link between the mitoNEET protein, peroxisome proliferator-activated receptor-y, and type 2 diabetes (T2D) (Biessels and Reagan, 2015; Colca et al., 2004). Following studies have shown diagnostic associations between not only mitoNEET and T2D (Kusminski et al., 2016), but also Parkinson's disease (Geldenhuys et al., 2014; Hammack et al., 2015), cystic fibrosis (Taminelli et al., 2008), Alzheimer's disease (Biessels and Reagan, 2015; Geldenhuys et al., 2014), cardiac function (Furihata et al., 2018), as well as deficits in mitochondrial oxidative stress (Kusminski et al., 2012; Mittler et al., 2019). Development of pharmaceuticals designed to target mitoNEET seems like a logical step in mitoceutical development. From this, treatments for the disorders would not only increase in robustness, but also have the potential to halt or even reverse the progression of these diseases.

### 4.2 Mitochondrial transplant

Mitochondrial transplantation is the most recent treatment for disorders involving mitochondrial function (Fuente-Muñoz and Arias, 2021; Lightowlers et al., 2020; McCully et al., 2016; Norat et al., 2020; Picone et al., 2020). In vivo, this idea is not new, as endogenous cell-cell transfer of mitochondria has been observed following cellular damage (Islam et al., 2012; Li et al., 2014). This is thought to be completed via nanotunnels between the cells, yet the mechanisms of transfer and the determination of which cell is the donor vs acceptor is not well understood (Chang et al., 2019).

Mitochondrial transplantation efforts have been utilized in rodent models of cardiac (Chernyak, 2020; Lim, 2020) and pulmonary ailments (Hsu et al., 2020; Li et al., 2014), with promising results in studies using a pulmonary hypertension model in adult rats (Hsu et al., 2020). Treatment of disorders, such as Alzheimer's disease, depression, and anxiety, using mitochondrial transplantation is of high interest. Mitochondrial transplant has been utilized in the treatment of neurodegenerative diseases, including Alzheimer's disease and Parkinson's disease, in rodent models (Fuente-Muñoz and Arias, 2021; Mukherjee et al., 2020; Norat et al., 2020). More recently, mitochondria nasal-sprays have been used in male and female mice to treat cisplatin-induced cognitive deficits (Alexander et al., 2021). Translationally, mitochondrial transplantation has not been a major focus for clinical applications. This may be due to a few reasons, notably lack of basic research to serve as a foundation supporting the development of translational studies as well as the lack of animal models that comprehensively display signs and symptoms seen within human disease. The utilization of healthy mitochondria to treat the effects of neurodegenerative disorders, as well as mitochondrial deficits resulting from natural aging in combination with hormone therapy is highly promising for the future of neurodegenerative and translational neurobiology research.

Use of this technique following traumatic brain injury (TBI) has also been explored. The determination of mitochondrial changes following TBI may provide foundational evidence for neurocognitive research. TBI is linked to an increased risk cognitive decline, mood disorders, and the development of dementias (Washington et al., 2016), with a recent study suggesting estrogen treatment minimizes detrimental cognitive effects following TBI in male mice (Wang et al., 2021; for review see Kövesdi et al., 2021). Mitochondrial dysfunction has been documented following TBI (Fischer et al., 2016; Normoyle et al., 2015; Pietro et al., 2017) and appears to act in a sex-specific mechanism (Greco et al., 2020). Male and female rats with a history of TBI show altered substrate utilization within the mitochondria, with females displaying no improvement when given the β-oxidation substrate beta-hydroxybutyrate (BHB) and males displaying mitochondrial improvement when the substrate lactate was administered immediately post and when BHB was administered 6 hours later (Greco et al., 2020). Transplantation of healthy mitochondria, in theory, may correct deficits in substrate utilization, yet more research needs to be conducted to reveal the root cause of this sex difference post TBI and if mitochondria can be transplanted from a donor of a different sex.

## 5 Mitochondria and the future of psychoneuroimmunology

It is evident that further research needs to be done on this topic to obtain a more comprehensive view of the complexities of these interactions and how they may be a key factor in early identification and treatment of various disorders. The overarching message is that mitochondrial function is dynamic and highly sensitive to modulations of steroid hormones, including estrogen. These hormonal changes influence not only energy production, but also play a major role in intracellular signaling, mitochondrial and nuclear gene transcription, and cell survival. Moreover, dysfunctional mitochondria may lead to gross alterations in neuroanatomy as well as drive behavioral presentation of many disorders.

Estrogen appears to balance mitochondrial responses to environmental insult, protecting the mitochondria and cell from its own demise. Increased focus on both hormone-related sex differences and mitochondrial modulations may open doors for the development of new therapeutics (Andrabi et al., 2017; Chang et al., 2019) (i.e. mitoceuticals) that may prevent the progression or development of behavioral and/or neurodegenerative disorders. The mitoceutical MitoQ, a compound based on coenzyme Q10 (CoQ10), is an antioxidant specifically targeted to the mitochondria (Tauskela, 2007), with a number of studies completed *in vitro* and *in vivo* in both rodents and humans (Smith and Murphy, 2010). Notably, *in vitro* and *in vivo* rodent studies have shown that MitoQ treatment minimized neurodegeneration caused by Aß-accumulation (McManus et al., 2011), prevented cognitive decline, and synaptic mitochondrial dysfunction (McManus et al., 2011; Olesen et al., 2020). Studies linking MitoQ to estrogen within the brain are limited, yet a study assessing GPER-mediated cardiac function suggests a connection exists. Here, female mice with a cardiomyocyte GPER-knockout were treated with MitoQ for 8 weeks. When compared to the non-treated knockouts, MitoQ treatment restored abnormal cardiac weight, UCP3, and NADPH oxidase 4 to near normal levels, in addition to other signs of restored cardiac function (Wang et al., 2018). Other mitochondrial-targeted treatments have been studied for use in cancer therapies (for review see Battogtokh et al., 2018), yet exploration of estrogen's role in mitoceutical function is sparse. Estrogen-mitoceutical interactions should be a key question in the future research and testing of these cutting-edge treatments.

Understanding the basics of estrogen-mitochondria interactions is only the first step. Complex interactions between estrogen, inflammation, and mitochondrial function are understudied especially within females. In a recent study by myself and colleagues (Shaw et al., 2021), results suggest that mitochondrial respiration within isolated synaptosomes are dependent upon estrogen levels. The mitochondrial response to the timing of an inflammatory challenge was dependent on the cycle stage at the initiation of LPS administration. Here, we found a baseline increase in synaptosomal mitochondrial respiration in female Wistar rats treated with LPS when compared to their saline treated, estrus stage matched controls. This difference was not seen between treatment groups of rats in diestrus. Likewise, between the controls, we observed an overall increase in synaptic mitochondrial respiration in the diestrus control group when compared to the estrus control group. Estrus cycle-dependent differences have been previously reported in various aspects of the neuron, from electrophysiological mini excitatory postsynaptic currents (Proaño et al., 2020) to alterations in the production of transcription factors within the medial prefrontal cortex of rodents (Duclot and Kabbaj, 2015).

The common factor between these studies is the interaction of estrogen within the nucleus and mitochondria. Grasping a better understanding of the mechanisms suggested here will enable further development of therapies aimed to combat age and estrogen related mitochondrial malfunction and may explain the complex interactions with cycle stage and inflammation that has been shown to yield cycle stage specific outcomes. The changes appear to occur in the mitochondria first, suggesting mitochondrial malfunction may be a prodrome to a variety of disorders (Apaijai et al., 2020; Araujo et al., 2020; Rettberg et al., 2014; Song et al., 2021). Because early detection is the key to longevity, it is of critical importance to study and understand the complexities of non-classical hormonal interactions within the mitochondria and how they alter the cell to create an environment susceptible to disease.

Taken together, mitochondrial research and treatment development is at the forefront of the future of basic, translational, and clinical research. Understanding the complex and sometimes sex-specific interaction between mitochondria and hormone receptor activation will open doors for a more personalized treatment schedule. From cancers (Mittler et al., 2019; Sastre-Serra et al., 2010) to adverse effects of hormone decreases as a result of normal aging (Anderl et al., 2020; De Wit et al., 2020; Hampson, 2020; Porcu et al., 2019; Sharma et al., 2020), mitochondria will be the therapeutic target of the future.

## Declaration of competing interest

The author, Gladys A. Shaw, has no conflicting interests or funding to report
